# Virtual landscape-scale restoration of altered channels helps us understand the extent of impacts to guide future ecosystem management

**DOI:** 10.1007/s13280-022-01770-8

**Published:** 2022-08-19

**Authors:** Siddhartho Shekhar Paul, Eliza Maher Hasselquist, Amanda Jarefjäll, Anneli M. Ågren

**Affiliations:** 1 Department of Forest Ecology and Management, Swedish University of Agricultural Science, Skogsmarksgränd, 901 83 Umeå, Sweden; 2 Department of Earth Sciences, Uppsala University, Villavägen, 752 36 Uppsala, Sweden

**Keywords:** Boreal landscape, Catchment area, Flow accumulation, LiDAR, Peatland, Soil

## Abstract

Human modification of hydrological connectivity of landscapes has had significant consequences on ecosystem functioning. Artificial drainage practices have fundamentally altered northern landscapes, yet these man made channels are rarely considered in ecosystem management. To better understand the effects of drainage ditches, we conducted a landscape-scale analysis across eleven selected study regions in Sweden. We implemented a unique approach by backfilling ditches in the current digital elevation model to recreate the prehistoric landscape, thus quantifying and characterizing the channel networks of prehistoric (natural) and current (drained) landscapes. Our analysis detected that 58% of the prehistoric natural channels had been converted to ditches. Even more striking was that the average channel density increased from 1.33 km km^−2^ in the prehistoric landscape to 4.66 km km^−2^ in the current landscape, indicating the extent of ditching activities in the northern regions. These results highlight that man-made ditches should be accurately mapped across northern landscapes to enable more informed decisions in ecosystem management.

## Introduction

Hydrological connectivity is a key factor for nutrient cycling, water quality and quantity, carbon sequestration, and biodiversity (Kuglerová et al. 2014; Laudon et al. 2016; Kritzberg et al. 2020). To what extent human modification has altered hydrological connectivity has implications for how the ecosystem management and related government authorities should manage wetlands and waterways in future, particularly in the age of climate change and the United Nations Decade of Restoration (https://www.decadeonrestoration.org/). Furthermore, understanding which types of wetlands and waterways have been impacted also provides goals or endpoints for restoration and helps to set priorities for which type should be targeted (e.g., the rarest; Palmer et al. 1997).

At a minimum, 15 million hectares of wetlands in northern boreal forests in Europe and parts of North America have been drained over the last century to improve forest productivity (Paavilainen and Päivänen 1995). These efforts have profoundly affected the forest dynamics and hydrology of this critical boreal landscape (Norstedt et al. 2021). During the last few decades, researchers have highlighted a myriad of environmental concerns associated with forest ditching in Sweden, especially for large carbon emissions from the drained peatland (Peacock et al. 2021b). As such, peatland restoration has been encouraged through state funding as part of Sweden’s efforts to achieve more sustainable forest and environmental management. Although it is clear that many ditches have been dug in peatlands (Päivänen and Hånell 2012), ditching has also been performed in an uncertain number of hectares of wet mineral or even dry soil (Hasselquist et al. 2018). In addition, ditches have likely impacted wetlands hidden beneath the forest canopy that encompass a few types of cryptic wetlands (Creed et al. 2003), like discrete riparian inflow points (DRIPs) (Ploum et al. 2018), and groundwater discharge areas (Kuglerová et al. 2014). These cryptic wetlands are small areas with saturated soils in a swale that often have an elongated shape and are found at the bottom of small valleys where groundwater flow paths converge. Such wetlands are identified as flushes or peat flushes by British practitioners (Yorkshire Peat Partnership 2020) while Swedish forestry professionals refer to them as “Surdråg” (Hannerz 2020); however, we will use the term ‘cryptic wetland’ in the manuscript.

Whether the water moves as diffuse groundwater flows in the pore volume of the soil or in a channel has major implications for hydrology, biogeochemistry, greenhouse gas (GHG) balance, and ecology. A channel allows the water to move faster than groundwater, affecting the residence times for water, resulting in shorter lag times and flashier storm hydrographs (Prévost et al. 1999). Groundwater is often supersaturated with GHGs, which are released into the atmosphere when the water enters a channel (Wallin et al. 2018). Stream CO_2_ and CH_4_ emissions from low-order streams (i.e., 1st to 4th order) in Sweden have been estimated to be 2.7 Tg C year^−1^, where around 70% of the total emissions were derived from 1st and 2nd order streams (Wallin et al. 2018). A recent study covering subtropical, temperate, and sub-arctic biomes identified 93% of the artificial water bodies (i.e., ditch and pond) as GHG sources (Peacock et al. 2021c) while another similar study reported CH_4_ emissions were significantly high from the artificial ditches in tropical biome (Peacock et al. 2021a). These GHG emissions per unit area were larger compared to the emissions from the natural water systems. Thus, small-scale channels are a dominant component of carbon export through the aquatic conduit of the boreal landscape.

The formation of a natural channel network is controlled by channel initiation, which is an important geomorphic process influencing various ecosystem functions. A channel typically refers to a linear depression of variable width that provides persistent flow paths for water movement, typically a stream or river (Allan et al. 2020). Natural channels are predominantly created by flowing water that starts at channel heads—the highest point upstream of concentrated water flow and sediment transport between definable banks (Dietrich and Dunne 1993). Artificial channels, on the contrary, usually do not follow the natural channel initiation process and are introduced by various anthropogenic activities, e.g., in the Swedish landscape through ditching or straightening of natural channels. Understanding the average initiation threshold for natural channels in low gradient landscapes is important for understanding how to manage them in future. Small headwater streams make up a greater proportion of the total stream length than larger downstream rivers (Benstead and Leigh 2012). For example, in Sweden, it has been estimated that 95% of the total stream and river length are low-order streams (stream order 1–4) (Wallin et al. 2018), but they are rarely present on maps (Ågren et al. 2015). A comprehensive understanding of how these low-order streams and the man-made ditches are distributed across the Swedish landscape will be pivotal for prioritizing ecological restoration. Yet, detailed information on the channel networks across the country is currently insufficient, and hence, their site-specific impacts on the hydrology and adjoining ecosystems are not clearly understood.

Maps were historically drawn from aerial photos; however, small-scale channels in the forest landscapes are often hidden beneath the canopy impeding their identification in the aerial photos. For example, our comparison with a national field survey and the Swedish topographical maps (1:12 500) shows that only 45% of the natural watercourses, 25% of the straightened channels, and 9% of the ditches (pertaining to channels < 6 m wide) are currently mapped in Sweden. High-resolution Light Detection and Ranging (LiDAR) measurements are now providing unique opportunities to map these missing channels in the Swedish landscape. In this study, laser scanning data with 1–2 points m^−2^ were used to generate a digital elevation model (DEM) at 0.5 m resolution. Small-scale channels can now easily be detected using such high-resolution DEMs. Moreover, these DEMs can also be utilized for accurately detecting channel heads, which marks the start of a longitudinally continuous channel downstream. Since the natural channel heads are related to the contributing drainage area, the detection of channel heads provides a means to model channel networks in a landscape.

The purpose of this study was to understand the extent of the human impacts on the hydrology and hydrological connectivity of the forest landscape to help guide future ecosystem management decisions. We asked the following questions: (1) How large of a catchment area is needed for a channel to be initiated naturally (i.e., channel initiation threshold)? (2) Are there regional differences between northern and southern Sweden? and (3) To what extent has the natural channel network been modified? To answer these questions, we manually digitized ditch networks in 11 selected forest regions in Sweden. Furthermore, we digitized the location of natural channel heads as well as transition points where natural channels connect man-made ditches. This allowed us to estimate the catchment area needed to erode a natural channel in this system. We used this channel initiation threshold to model a “prehistoric” channel network. Here we use the term “prehistoric” to define a landscape before humans altered the flow paths of water in the landscape, the timing of which depends on where you are in Sweden. Initially, most drainages were performed for agricultural production and with dominance in southern Sweden where most of the population lived (Jakobsson 2013). But with the colonization of northern Sweden, ditches were initially (1600–1700s) dug for hay production while ditching for forest production took over in the 1900s (Norstedt et al. 2021). By comparing the current channel network with the “prehistoric” network, we quantified and characterized the man-made alterations of the channel networks across the 11 regions. We hypothesized that—(1) a large range in catchment areas or channel initiation threshold would be needed to initiate a natural channel; (2) the proportion of modified waterways would increase from north to south, corresponding to the human population levels in Sweden, though forest ditches would be more prominent in the northern regions; and (3) the channel network has been significantly lengthened by humans and differences between the hydrological connectivity of the natural channels and man-made channels would be significant. Finally, we discuss the implications of the results of the study to improve environmental management.

## Materials and Methods

### Study area

Sweden (latitude 55–70° N, longitude 11–25° E) is a forest-dominated country in Northern Europe. Seventy percent of the country’s landmass is forest (Felton et al. 2020). Sweden is sitting primarily on quaternary glacial till deposit (Lundqvist et al. 2004). Although at high latitude, Sweden is characterized by a temperate climate with distinct seasonal variation and somewhat mild temperature. There is a notable elevation and precipitation gradient from north to south of the country, with annual precipitation ranging from 400 to 2100 mm (1961–1990).

**Fig. 1:**
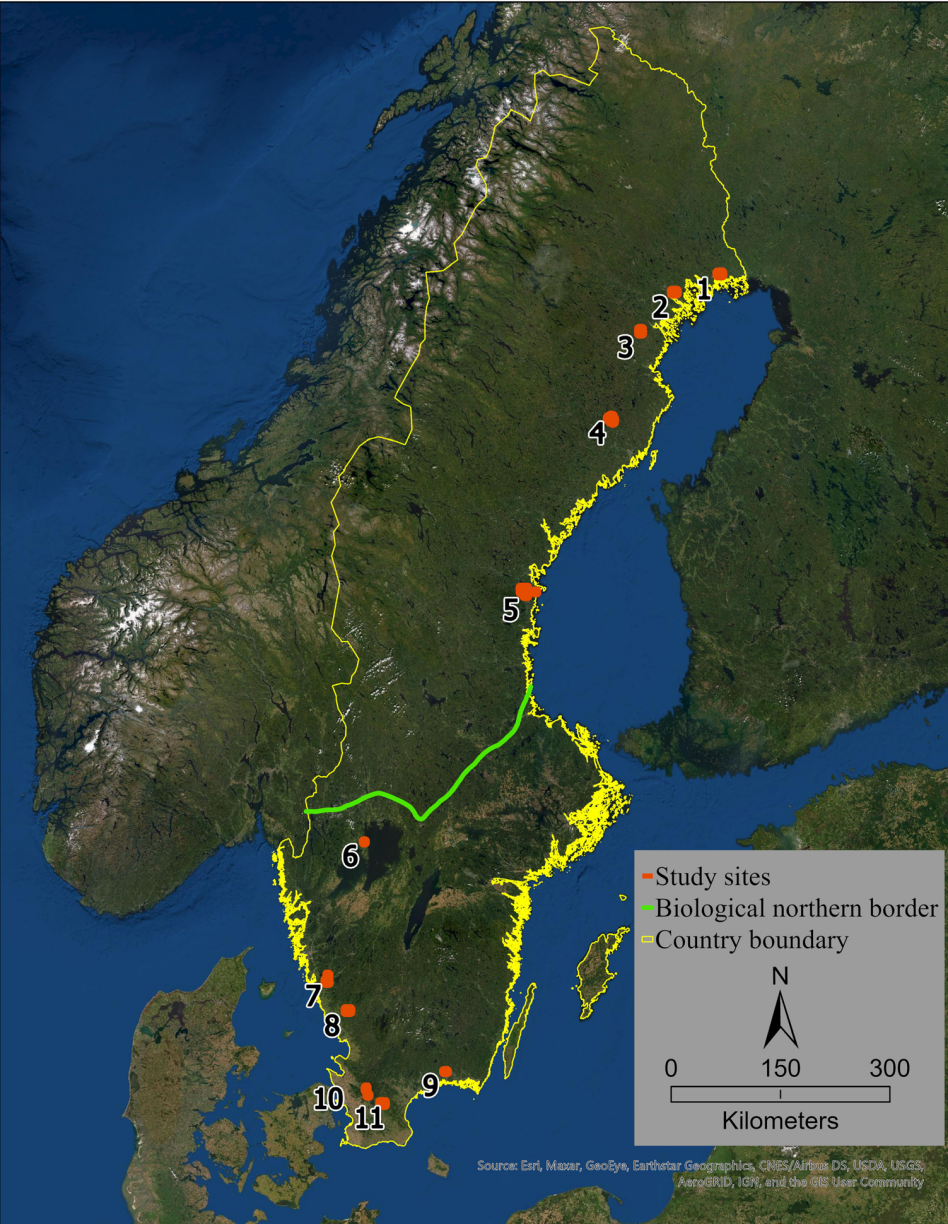
The study regions (*n* = 11) across Sweden. Region 4 is the Krycklan catchment—the long-term research site managed by the Swedish University of Agricultural Sciences. Regions 1–5, which are north of the biological northern border or “Limes Norrlandicus” (Gullefors 2008) were reported as ‘northern study regions’ in this analysis while regions 6–11 were designated as ‘southern study regions’

Our study included 11 regions across Sweden (Fig. 1), among which Krycklan catchment (68 km^2^) is a long-term research site managed by the Swedish University of Agricultural Sciences (Laudon et al. 2021). The remaining 10 regions were selected based on the following characteristics: (i) land cover type is predominantly forest, (ii) the regions span across the country from north to south and comprise a wide variability for topography, soil, runoff, tree species, etc., and (iii) the regions fall within the ongoing national Swedish laser scan for producing high-resolution DEM. The 11 study regions were divided into northern study regions (i.e., regions 1–5) and southern study regions (i.e., regions 6–11) based on the biological northern border or “*Limes Norrlandicus*,” which divides the northern boreal forests and southern boreonemoral areas of Sweden (Gullefors 2008, Fig. 1).

### Airborne laser scanning (ALS)

We utilized 55 LiDAR images of 2.5 × 2.5 km each (total of 344 km^2^) in addition to the Krycklan Catchment (68 km^2^) for channel network detection (Fig. 1). The landscape was scanned from an elevation of 3 000 m using a scanning angle of ± 20º. The generated point cloud had a point density of 1–2 laser points m^−2^, with a vertical accuracy of 0.1 m and a planar accuracy of 0.3 m. Up to 7 echos were registered and the last return was used to generate a digital elevation model of the ground surface with 0.5 × 0.5 m resolution.

### Mapping of ditch networks

**Fig. 2:**
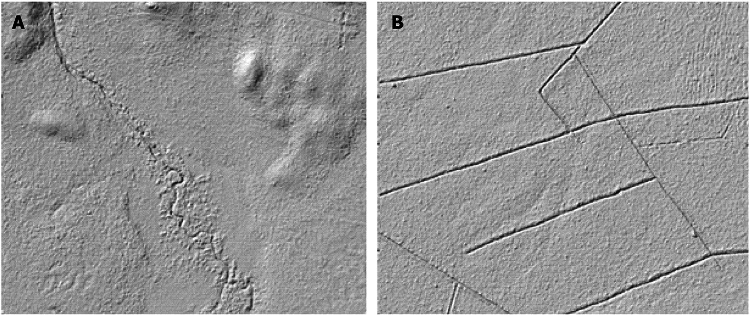
Appearance of (**A)** natural channels and (**B)** man-made ditches as viewed on the hillshade derived from 0.5 m digital elevation model (DEM). The natural channels are typically meandering and give a “fuzzy” impression on the DEM. However, ditches usually exhibit a straighter and smoother impression than natural channels

The ditch networks were manually digitized as vector lines by a group of trained professionals at the Swedish Forest Agency. A hillshade with a Z-factor of 2 (to enhance the elevation differences between channels and the surrounding landscape) was generated from the 0.5 m DEM. A High Pass Median Filter (HPMF) was also calculated using a kernel of 11 cells to separate local ridges from local depressions (e.g., channels). When distinguishing ditches from streams, we considered the degree that they were straightened, smoothened edges, and obvious mounds on the sides of channels. Natural channels are typically meandering and even if they are relatively straight in some locations, we observed that natural channels give a “fuzzy” impression in the DEM (Fig. 2A). This is likely because the stones and boulders were not artificially removed from the natural channels. However, artificial ditches (Fig. 2B) are generally straighter with a smoother appearance in the DEM, likely because the gravel and small borders were manually removed from the channels and form the small mounds found along the sides of the ditches.

**Fig. 3:**
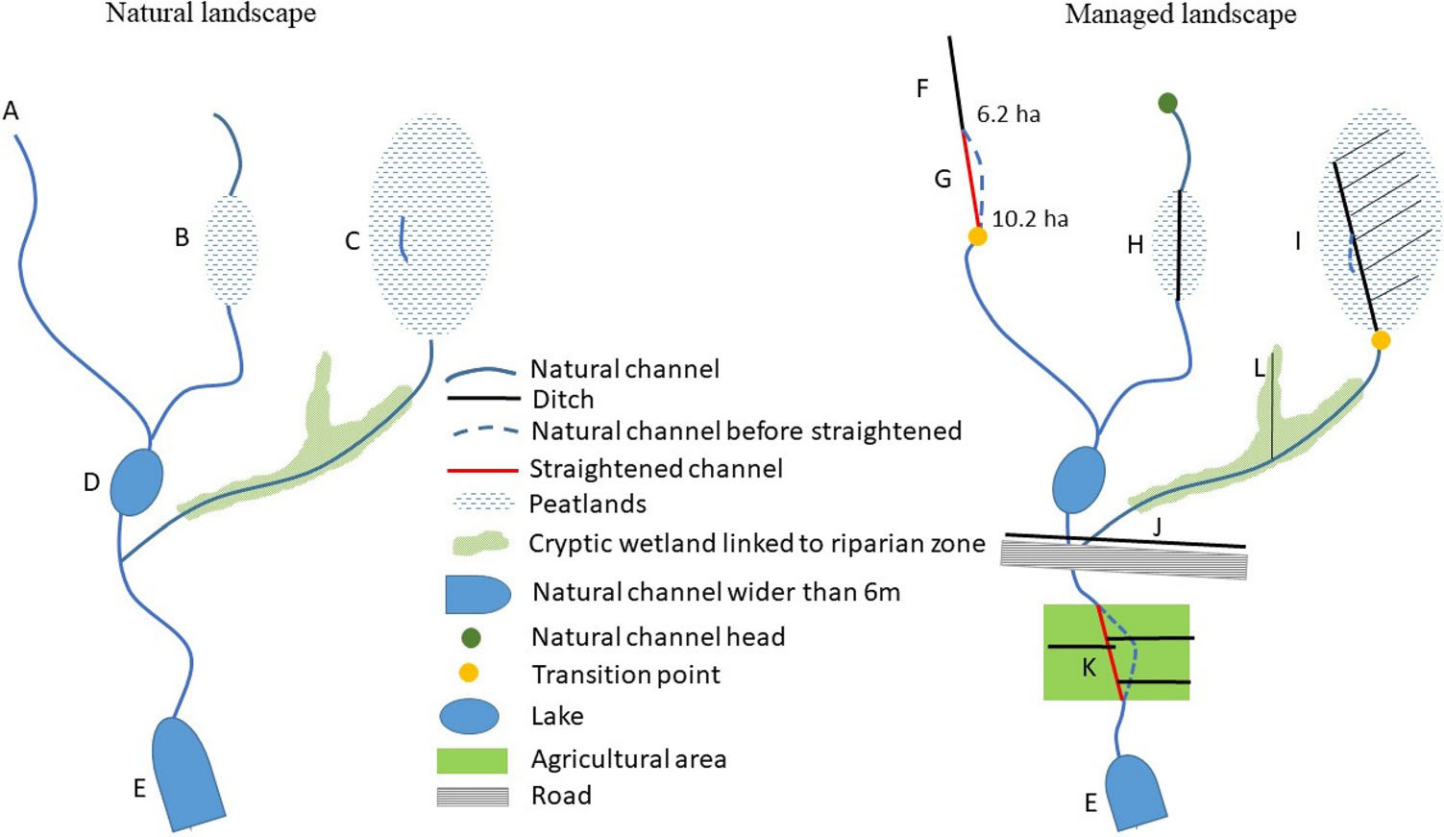
Conceptual diagram showing the components of natural and man-made channel networks in Sweden

In addition to hillshade and HPMF, historical and current orthophotos, land use maps (present and historical) were used to distinguish the natural channels and man-made ditches. When digitizing the ditches, the DEM was first zoomed out to get an overview; then it was zoomed into a scale of 1:600 to ensure the digitized line fell inside the ditch. The national property map of Sweden (1:12 500) was used to classify the digitized ditches into agricultural, forest, and road ditches. Road ditches existed on one or both sides of the roads and follow the road network, so they can cross all landscapes and drain both wet and dry soils (Fig. 3J). In some cases, the natural channels were straightened (Fig. 3G, K), which was more obvious in agricultural areas than forested areas; for example, natural channels were straightened to align with the agricultural ditch networks in Fig. 3K. Forest ditches were often found at the bottom of small valleys and were often connected to natural channels (Fig. 3F). Ditches were also dug in the cryptic wetlands (Fig. 3L), which in many cases are linked to the riparian zones of the natural channels. In some cases, forest ditches were old natural channels that had been straightened and deepened (Fig. 3G), but mostly, they have been dug upstream of the natural channels to elongate the channel network (Fig. 3F). Large peatland areas also had forest ditches with variable densities (Fig. 3H, I).

### Mapping current natural channel network

The mapping of natural channels was conducted in three steps—(i) detecting all natural channel heads, (ii) tracing the downstream channels from the heads, and (iii) manual editing of the channels. First, we manually traced each natural channel upstream on the DEM to locate the points where an incised natural channel was no longer detectable. This point could either be a natural channel head (green point in Fig. 3) or the point where the natural channel connects to an upstream ditch system (yellow point in Fig. 3), which is termed as ‘transition point’ in this article. The variability in contributing catchment area between natural channel heads and transition points was tested with a nonparametric statistical test—the Wilcoxon test (Hollander et al. 2013). To map the channel networks we first prepared the 0.5 m DEM according to the best preprocessing method for the Swedish boreal landscape (Lidberg et al. 2017) by burning streams across roads and breaching depressions to create a flow compatible and hydrologically correct DEM. We then snapped the digitized channel heads and transition points to the closest flow accumulation cell with a search radius of 10 m. From each channel head/transition point, the downstream natural channels were mapped using the algorithm ‘Trace Downslope Flowpaths’ in Whitebox Tools (Lindsay 2018). This utilizes the D8 flow accumulation algorithm (O’Callaghan and Mark 1984), a non-dispersive algorithm that is particularly suited to watershed delineation and channel mapping (Lindsay 2018).

In this study, we focus on small channels of < 6 m in width. The lakes (Fig. 3D) and large channels > 6 m in width (Fig. 3E) were removed using a mask of lakes and large channels from the Swedish property map. In addition, the derived stream networks were manually edited when a channel could not be visually detected on the DEM. For example, peatland areas usually lack clear channels (Fig. 3B) as water moves as diffuse flow in the pore space (Rezanezhad et al. 2016) or follows a preferential flow layer underneath the mire surface (Fig. 3C, Holden and Burt 2003). Some sections showed an overlap between the automatically derived stream network and downstream ditch systems (Fig. 3K). Such sections were removed from the current natural channel layer and included with different ditch types.

### Modeling prehistoric natural channel network

**Fig. 4:**
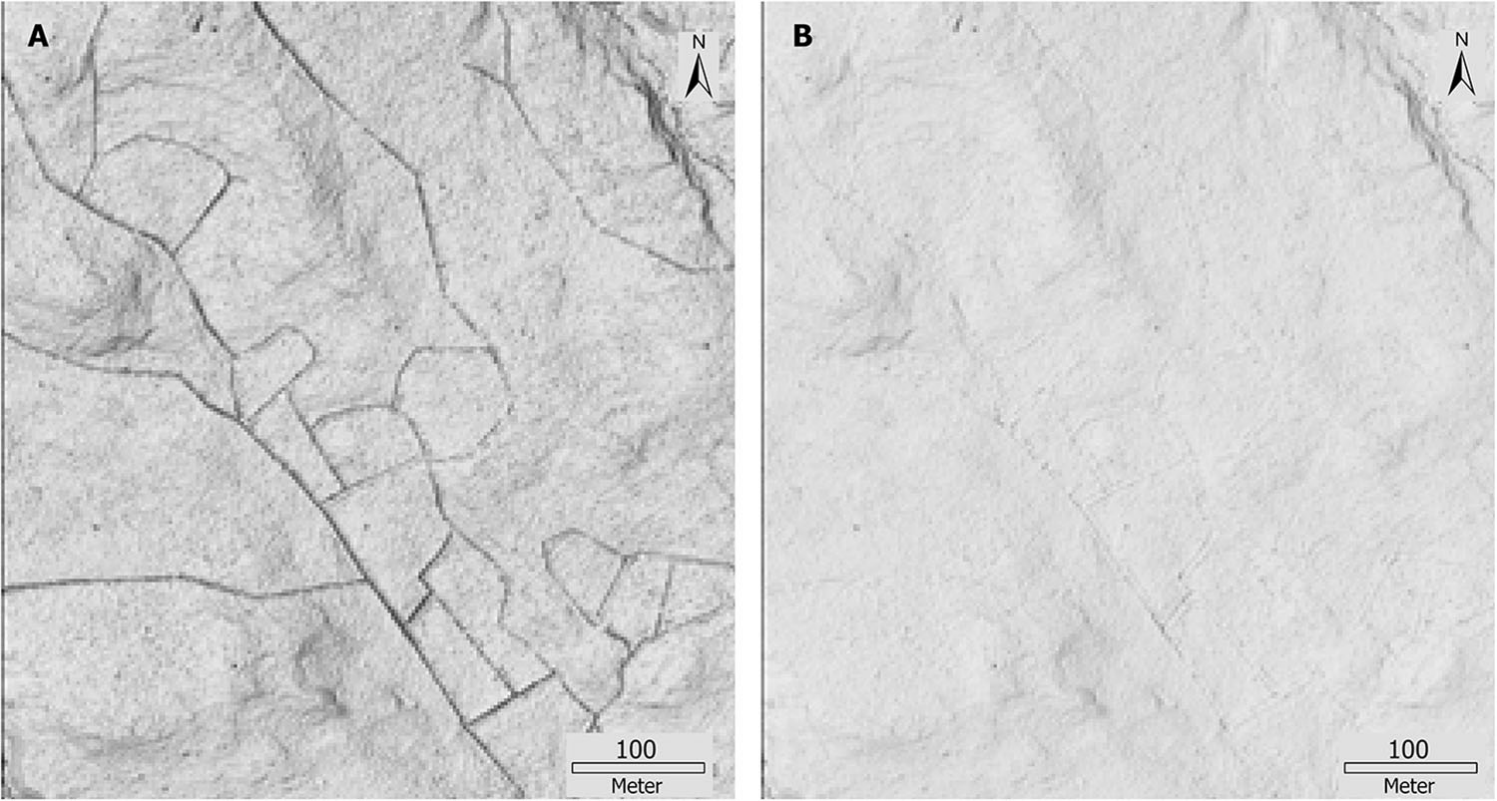
An example of a small area for the hillshade (with Z-factor 2) of the digital elevation model (DEM) showing the (**A)** current landscape with ditches and (**B)** modeled ditch-filled prehistoric landscape

**Fig. 5:**
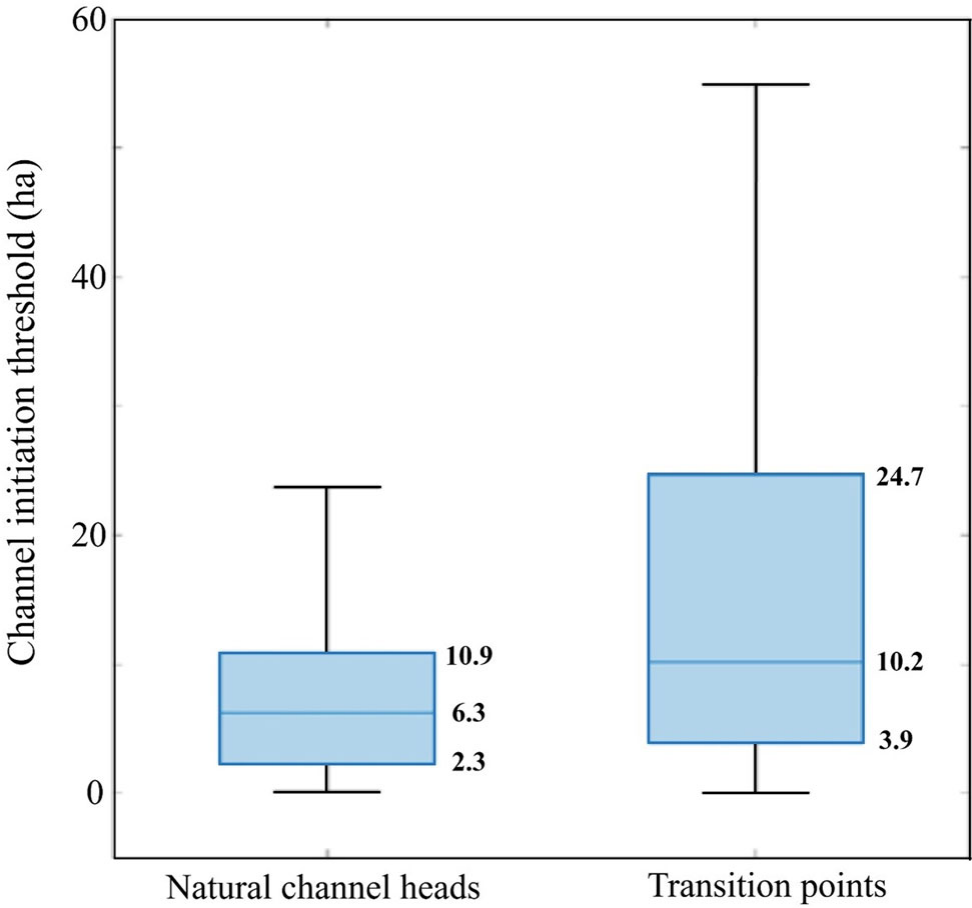
The distribution of the channel initiation threshold of natural channel heads (green dot in Fig. 3) and the transition points where natural channels connect to upstream ditch networks (yellow dot in Fig. 3). The extent of the box shows the 25th, 50th, and 75th percentiles of channel initiation thresholds (with values shown), while the error bar represents the 10th and 90th percentile

To model the prehistoric landscape, we digitally back-filled the man-made ditches by using the ‘Impoundment Size Index’ algorithm in Whitebox Tools (Lindsay 2018). We applied a dam length of 6 m (as we focused on channels < 6 m width) and extracted the dam height. The dam height layer was masked using a 4 m buffer around the digitized ditches to extract the depth of the ditches. By adding the elevations of the masked dam height to the original DEM, a new DEM was generated that provided the prehistoric landscape (Fig. 4A, B). The prehistoric channels were then mapped using the same approach as the current natural channels (regarding preprocessing and flow accumulation method) except that we used a fixed flow initiation threshold to map the stream network based on the upslope area of natural channel heads (i.e., 6.3 ha, Fig. 5). To estimate the uncertainty in the modeled prehistoric stream network, we also apply flow initiation thresholds based on the 25th and 75th percentile initiation values (i.e., 2.3 ha and 10.9 ha, Fig. 5). We manually edited this modeled prehistoric channel network for lakes, large streams, and diffused wetland flow in the same way as for the current natural channels (“Mapping current natural channel network” section).

### Hydrological connectivity of the natural channels and forest ditches

We explored the hydrological connectivity of current natural channels and forest ditches using flow accumulation. We located the channels using the same D8flow accumulation algorithm as in the trace downstream flow-path analysis in “Mapping current natural channel network” section. However, to ensure a better representation of the flow accumulation values, we burned the natural channel and ditch networks into the DEM at a depth of 0.5 m. To ensure that the “water flows inside the channels,” the burning of the networks was performed using a decay function that creates a slope toward the channels (Lindsay 2016). Multiple decay coefficients were tested for burning the channel network into DEM, and our expert judgment found a coefficient of 0.9 to route the water inside the channels without changing the topography too far away from the channels which may cause the water to flow in the wrong direction. Next, the DEM was prepared for calculating flow accumulation as described in “Modeling prehistoric natural channel network” section and the D-infinity flow accumulation algorithm (Tarboton 1997) was applied. D-infinity is assumed to model dispersive groundwater flow in a better way than D8, while our burning of the channels still ensures that this “groundwater is correctly routed into the channels” (Lindsay 2018). We then created points at every 0.5 m distance along the natural channels and ditch networks to extract values from the 0.5 m resolution flow accumulation raster. The extracted flow accumulation values were then summarized into 20 groups for both natural channels and forest ditches to identify the flow variation. We applied the Wilcoxon test to evaluate whether the flow accumulation differs between natural channels and ditches.

## Results

### Characteristics of channel heads

Approximately 150 person-hours were used to map 394 channel heads during the digitization process, where the majority (58%) were classified as natural heads and the remainder as transition points. Among the natural channel heads, 64% were in the northern study regions and 36% were in the south. However, transition points were more prominent in the southern study regions (56%) compared to the northern regions (44%).

As hypothesized, the channel initiation threshold reveals a large variability for both natural channel heads (0.1–58 ha) and transition points (0.05–193 ha). The mean threshold for the natural channel heads was significantly smaller than for the transition points (*p* < 0.001; Wilcoxon test) with a median of 6.3 ha compared to 10.2 ha (Fig. 5).

### Current and prehistoric channel network

**Fig. 6:**
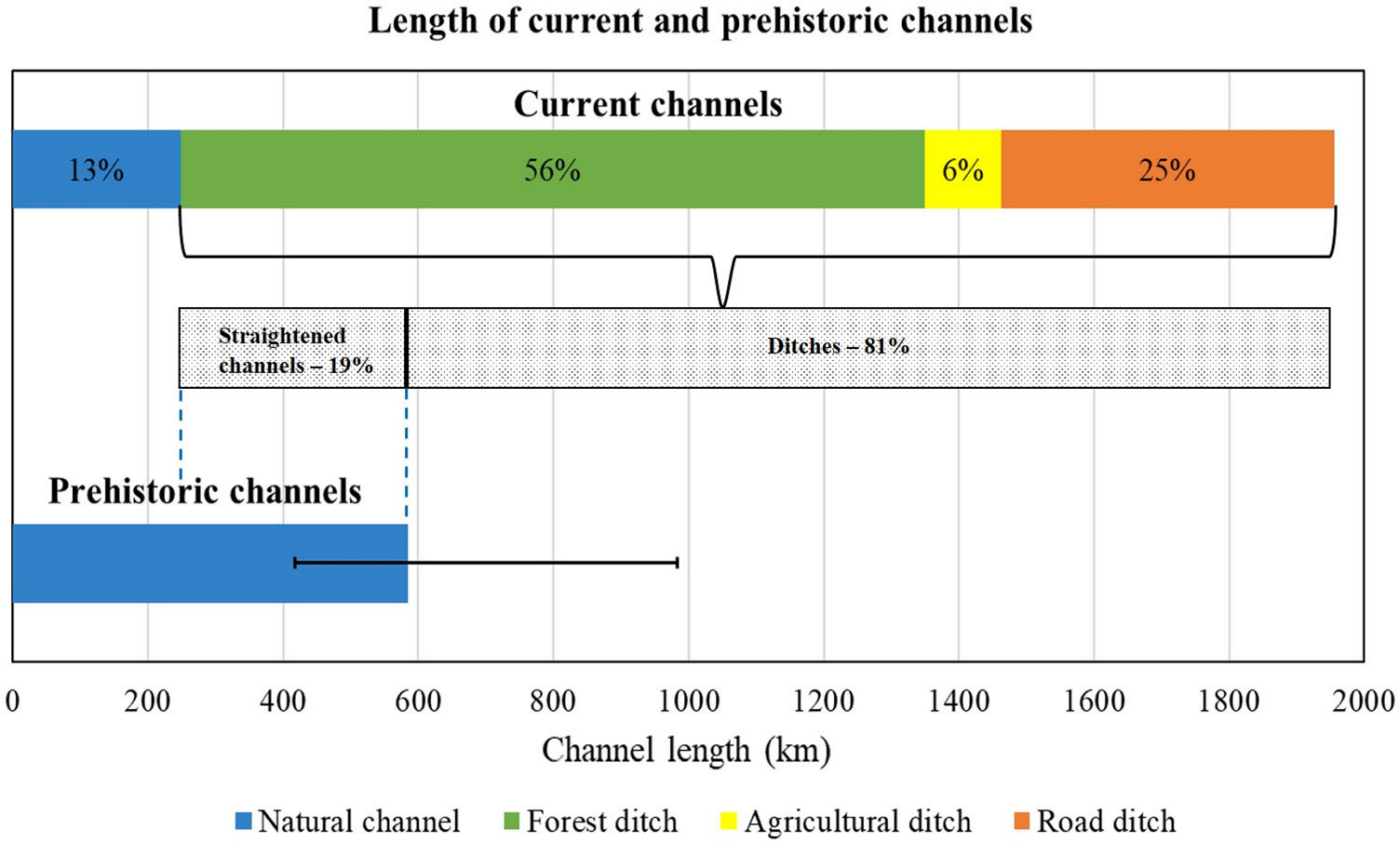
Total lengths of different channel types for the 11 study regions combined. The prehistoric channel length is modeled using the 50th percentile of the initiation threshold for manually mapped natural channel heads (i.e., 6.3 ha), with error bars based on models using the 25th and 75th percentiles. The current natural channels are generated using the “trace downstream flow paths” algorithm (Lindsay 2018) based on all digitized channel heads and transition points, then manually edited as described in “Mapping current natural channel network” section. The ditch lengths are based on manual digitization using a 0.5 m resolution digital elevation model

We mapped and categorized a total of 1955 km of channels within the current network in the 11 regions that we studied across Sweden. This network is dominated by man-made channels; with 87% of the current channel network modified by human activities and only 13% of them left as natural (Fig. 6, top bar). Of the man-made channels, most are forest ditches (56%), followed by road ditches (25%), and agricultural ditches (6%) (Fig. 6, top bar). In the reconstructed prehistoric landscape, we modeled 584 km of natural channels using the 50th percentile of the channel initiation threshold (6.3 ha, Fig. 6, bottom bar). However, due to the variability in flow accumulation thresholds of the natural channel heads we mapped, the prehistoric channel length could have been between 417 and 984 km when initiation thresholds of 2.3 ha (i.e., 25th percentile) and 10.9 ha (i.e., 75th percentile), respectively, were applied.

With all regions combined, we found that 58% of the prehistoric natural channels have been straightened. In another word, 19% (ranging between 10 and 43% when 25th and 75th percentile channel initiation thresholds were considered) of the current ditch network was once part of the natural channel network (Fig. 6, middle bar) and the remaining 81% (ranging between 57 and 90% when 25th and 75th percentile channel initiation thresholds were considered) of the ditch network was dug to extend the network upstream (Fig. 6, middle bar).

**Fig. 7:**
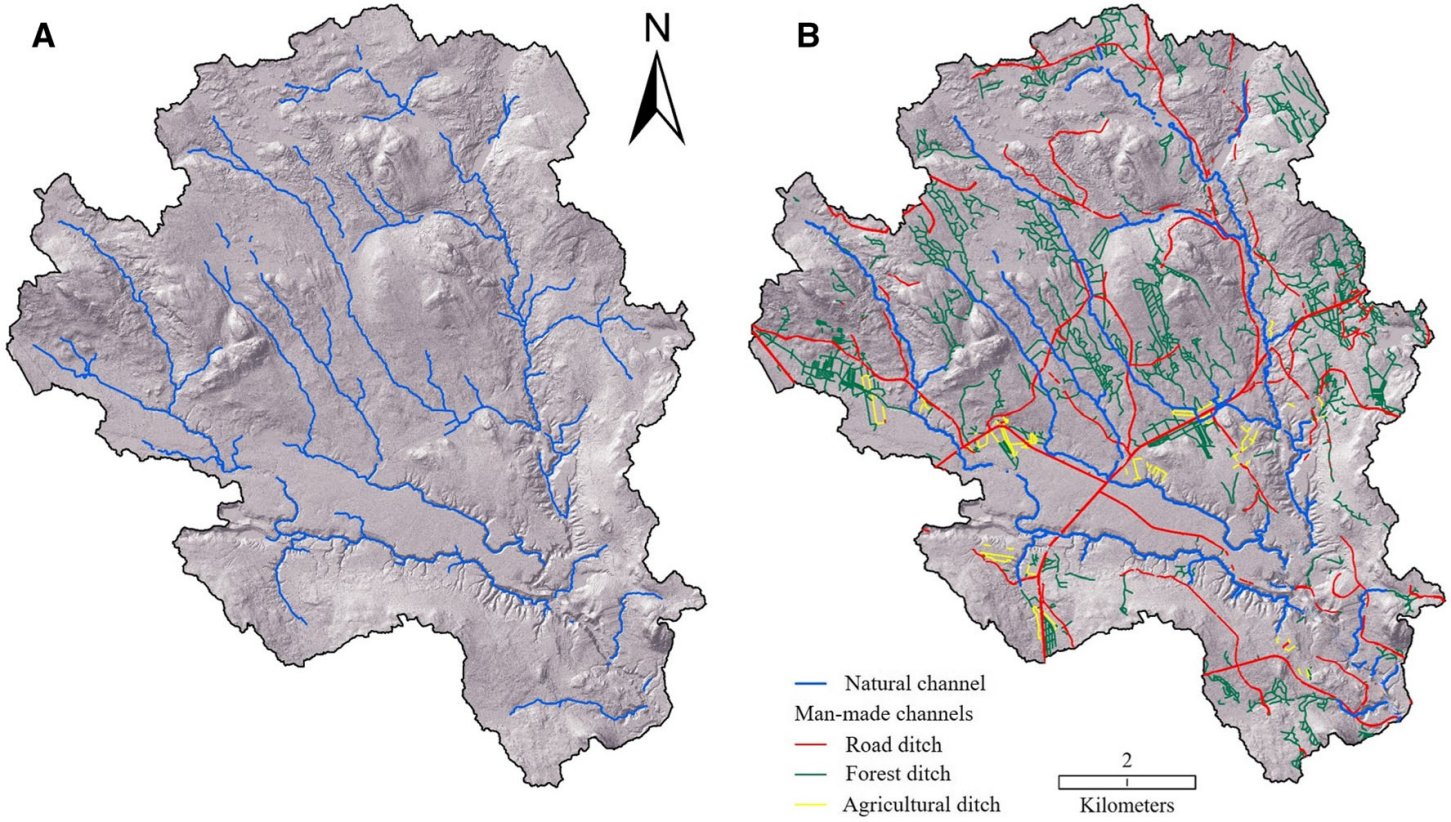
**(A)** The prehistoric natural channel network of the Krycklan Catchment (region #4). The network is modeled using a 6.3 ha channel initiation threshold, based on the digitized natural channel heads (Fig. 5). (**B)** The current channel network of the Krycklan Catchment. The ditch network was manually digitized using a 0.5 m digital elevation model while the natural channels were generated using all digitized channel heads and “trace downstream flow paths” algorithm (Lindsay 2018). For both (**A**) and (**B**), the wider channels near the outlet, which are > 6 m wide, as well as lakes and diffuse flow in the peatlands were removed

In the prehistoric modeled landscape, the average natural channel density for the studied regions was 1.33 km km^−2^. However, currently, the average total channel density (i.e., natural and man-made) is 4.66 km km^−2^ (Table 1), which agreed with our hypothesis that human modification had substantially lengthened the channel network (see Fig. 7 for an example study region where we displayed the increase in channel density). The average density of current natural channels is 0.48 km km^−2^, just 36% of the total channel density in prehistoric landscape and 10% of the total channel density in the current landscape. The channel networks display a larger variability among regions in the current landscape (Table 1). Overall, the density of all channels decreased from the northern (5.07 km km^−2^) to southern (4.32 km km^−2^) study regions. Likewise, the density of all ditches was higher in the northern regions (4.50 km km^−2^) compared to the southern regions (3.92 km km^−2^). Therefore, our hypothesis that the south would have more modified waterways than the north, reflecting higher population densities, was not supported. However, the hypothesis that the forest ditches would be more numerous in the northern regions was supported; forest ditch density was 33% higher in the northern study regions than in the southern regions. In contrast, the density of agricultural and road ditches was higher in the southern study regions.

**Table 1:** Length and density of different categories of channels in our study regions across Sweden. The regions are roughly oriented from north to south, see Fig. 1 for specific locations. The total length includes both natural channels and ditches. The straightened channels are included with the ditches

Study regions (Northern or Southern, i.e., N or S)	Latitude	Total area (km^2^)	Prehistoric channels (with stream initiation threshold of 6.3 ha)		Current channels						
			Density—natural channel (km/km^2^)	Total channel length (km)	Density—natural channel (km/km^2^)	Density—agricultural ditch (km/km^2^)	Density—forest ditch (km/km^2^)	Density—road ditch (km/km^2^)	Density—all ditches (km/km^2^)	Density—all channels (km/km^2^)	Total channel length (km)
Region 1 (N)	65° 52′ 59″ N	37.50	1.38	51.77	0.19	0.08	4.13	1.14	5.35	5.55	207.95
Region 2 (N)	65° 43′ 46″ N	37.50	1.53	57.41	0.42	0.00	2.69	0.44	3.13	3.54	132.92
Region 3 (N)	65° 17′ 47″ N	37.50	1.61	60.43	0.53	0.00	6.54	0.69	7.23	7.76	290.96
Region 4 (N)	64° 15′ 6″ N	68.00	1.64	111.44	0.97	0.15	2.34	1.58	4.07	5.05	343.10
Region 5 (N)	62° 11′ 32″ N	106.25	1.10	116.60	0.73	0.46	1.09	1.19	2.73	3.46	367.33
Region 6 (S)	59° 6′ 35″ N	6.25	1.24	7.74	0.46	0.79	3.79	2.37	6.95	7.40	46.28
Region 7 (S)	57° 23′ 56″ N	31.25	1.08	33.89	0.24	0.26	2.52	1.74	4.52	4.77	148.94
Region 8 (S)	57° 01′ 43″ N	37.50	1.26	47.43	0.89	0.11	2.44	1.17	3.72	4.61	172.80
Region 9 (S)	56° 17′ 41″ N	12.50	1.04	12.95	0.30	0.48	0.63	0.19	1.31	1.61	20.11
Region 10 (S)	56° 02′ 55″ N	18.75	0.93	17.48	0.26	0.36	1.63	0.78	2.78	3.03	56.87
Region 11 (S)	55° 53′ 48″ N	37.50	1.79	67.31	0.25	0.58	2.50	1.15	4.23	4.48	168.16
Average density (N)			1.45		0.57	0.14	3.36	1.01	4.50	5.07	
Average density (S)			1.23		0.40	0.43	2.25	1.23	3.92	4.32	
Average density (All)			1.33		0.48	0.30	2.75	1.13	4.18	4.66	
Grand total				584.44							1955.43

### Hydrological connectivity of the natural channels and forest ditches

**Fig. 8:**
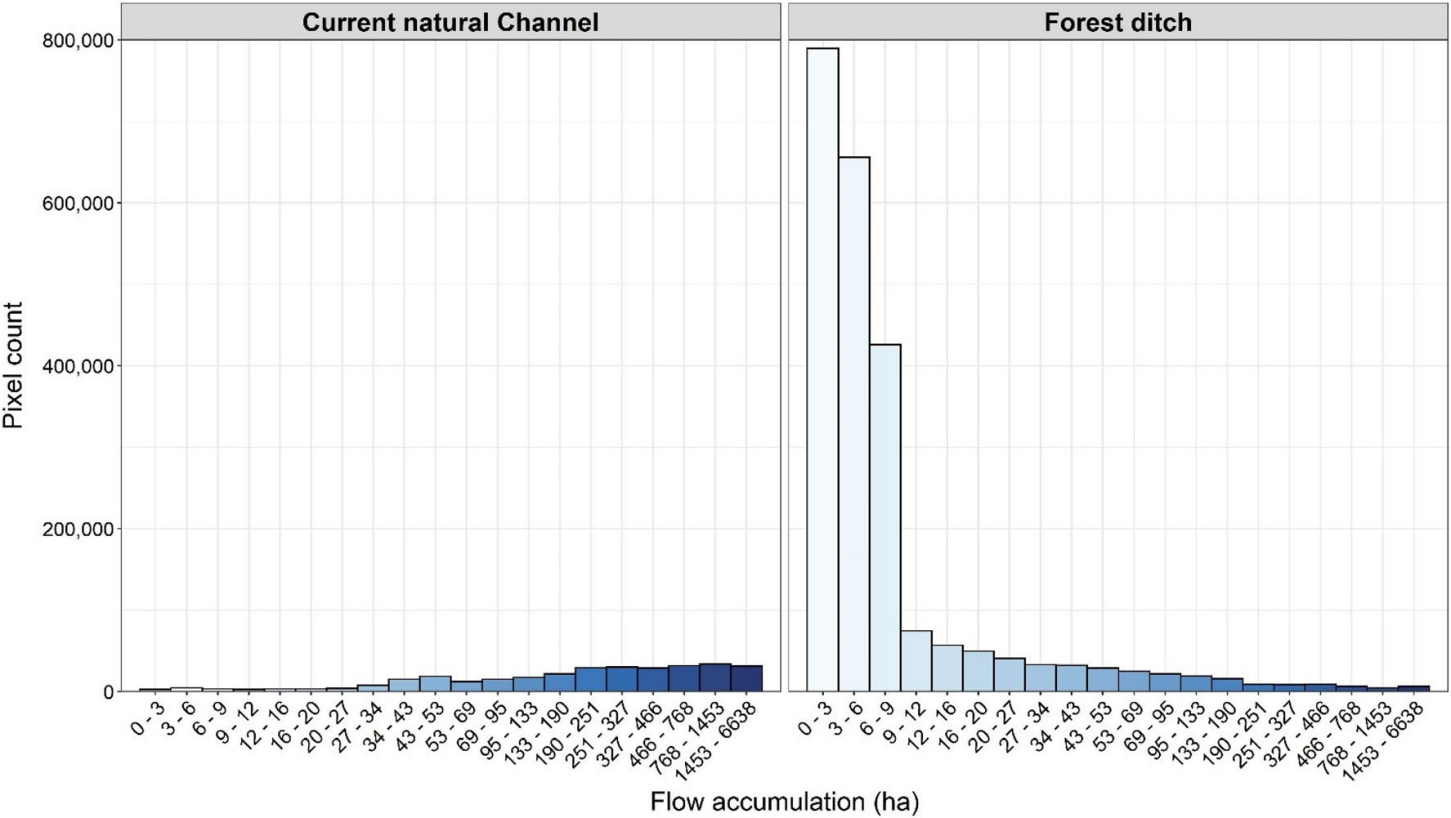
Histogram of the number of pixels of each flow accumulation class within the current natural channels (left) and forest ditches (right) derived from the 0.5 m resolution flow accumulation raster

As artificial ditching predominantly modified forest lands, our analysis explored the hydrological connectivity of the natural channels and forest ditches. Natural channels had significantly larger flow accumulation values than forest ditches (*p* < 0.001; Fig. 8); supporting our hypothesis that the hydrological connectivity of the natural channels and man-made channels would be significantly different, at least in forests.

We estimated that 90% of the natural channel pixels across our 11 study regions have perennial flow while the rest of the natural channel network is characterized by intermittent water flow using a threshold of 16 ha (Tiwari et al. 2016). In the case of forest ditches, just 13% of the ditch pixels were estimated to sustain perennial flow. This estimation of perennial water flow across the country will likely vary depending on the site characteristics and should not be taken literally, but it still indicates the extent of the hydrological connectivity of the different channel networks.

## Discussion

Although Swedish forests are generally considered remote and relatively unimpacted by humans, the most striking result of our study was the extent of the human modification of the channel network within the Swedish forest landscape by widespread ditching practices. Humans have more than tripled the channel length density than what likely existed in a prehistoric landscape (Fig. 6). These modifications have likely not just affected the hydrology and hydrological connectivity, but also their surrounding landscapes (i.e., as presumably intended, increased forest cover or reduced mire coverage). We estimated that 58% of the prehistoric natural channels have been straightened and thus, converted to ditches (Fig. 6). Today, these straightened channels make up 19% of the ditch network but the majority (81%) of current ditches were created by elongating the upstream channel network (Figs. 6 and 8). Forest ditches made up the majority of the man-made channels in the current network (56%). Much of this ditch network drained areas that previously had diffuse flow, i.e., DRIPS, but might also have drained relatively drier mineral soils (see Fig. 7 for changes between prehistoric and current landscape in an example region). Moreover, the drainage of peatlands and wet forests to increase forest production was also considerable. The altered natural channels were primarily straightened for timber floating (Laudon et al. 2021) and the elongation of the network to increase forest productivity, haymaking, and crop production; however, in most cases, this did not allow expert hydrological investigation and landscape evaluation (Norstedt et al. 2021). Additionally, even when expert advice did become available (Lundberg 1914), it was not necessarily followed (Hasselquist et al. 2018). Regardless, many of these historic activities have been or can be reduced or managed differently, and up to two-thirds of the current channels could be ecologically restored to return hydrological connectivity and ecosystem function.

Even with the high density of ditches in the Swedish landscape (currently, 4.18 km km^−2^ of ditches vs. 0.48 km km^−2^ of natural channels), in general, they are extremely under-researched when compared to natural streams (Koschorreck et al. 2020). We do know that ditch networks intercept and divert substantial quantities of groundwater and overland flow to stream networks, contributing to reduced water residence times and flashier flow characteristics (Buchanan et al. 2013). Therefore, ditches greatly increase the magnitude of peak flow and total event flow in downstream environments (Buchanan et al. 2013). This change in hydrology through drainage reduces in-stream processing of nutrients due to reduced residence times of water in the channel network and can affect the physical properties of the surrounding soils such as soil morphology, moisture levels, redox potentials, and overall biogeochemistry (Hayes and Vepraskas 2000). The lowering of the water table caused by ditching can increase the aerobic decomposition of organic matter, which may result in peat subsidence and compaction (Heikurainen 1957). Päivänen and Hånell (2012) suggested that in drained peatlands, aeration promotes microbial processes and solubility, and thereby increases peat mineralization, resulting in the increasing DOC concentration. This increase in peat decomposition level due to drainage, combined with naturally fluctuating water tables, increases loads of DOC and nutrients from drained peatlands (Nieminen et al. 2018). In addition, the increased bulk density of peat can substantially affect the water retention ability and hydraulic conductivity of the peat material (Minkkinen and Laine 1998; Laine et al. 2006). Moreover, drainage ditches have been identified as hotspots of GHG emissions (Hyvönen et al. 2013; Luan and Wu 2015), but the character of their GHG emissions depended on their intermittency (being wet or dry).

Giving more attention to larger rivers for environmental management activities has meant that intermittent and ephemeral channels, such as ditches, have received inadequate attention across the globe even though they constitute the world's most widespread river network (Messager et al. 2021). Therefore, man-made ditches should be carefully considered when deciding on appropriate ecosystem management (e.g., whether ditches should be cleaned or ecologically restored). Such ditches are characterized by low flow accumulation values (Fig. 8), so they have a significant influence on hydrology and biogeochemistry, affect landscape ecology, and provide habitat and other functions for streams (Clifford and Heffernan 2018).

Previous ditch detection studies have been performed mostly in open areas, such as marshes (Rapinel et al. 2015), grasslands and peri-urban areas (Roelens et al. 2018b), agrarian landscapes (Passalacqua et al. 2012; Cazorzi et al. 2013), and areas near roads (Kiss et al. 2015). In such open and easily accessible landscapes, ditch detection is relatively less complex as they rarely contain tree cover and ditches are usually well defined. However, we mapped a detailed network across an extensive landscape. By manually digitizing each ditch and channel head, we have prepared an accurate map of a small-scale channel network conducted for a large forested landscape to enhance the understanding of channel formation in these systems. Previous ditch studies utilizing LiDAR-derived DEM have mapped smaller areas up to 150 ha (Cazorzi et al. 2013; Rapinel et al. 2015; Roelens et al. 2018a) while we mapped 1708 km of ditches and 248 km of natural streams in 11 study regions covering a total of 415 km^2^. Only recently, Bhattacharjee et al. (2021) conducted a ditch detection analysis in a large forested peatland in Finland; this study was highly reliant on aerial imagery, and notable uncertainties were reported when aerial images were unavailable for parts of the study area. While the strength of manual interpretation of high-resolution DEM for ditch detection was demonstrated in this study, we also highlight the extensive investment of man-hours for manual digitization. Therefore, applying this methodology to even larger landscapes may be less feasible. But our dataset and mapping outcomes can be used to develop and evaluate automated methods for national-scale ditch detection and channel network mapping from LiDAR DEMs. Our study has highlighted that the intensity of channel network modification decreased from north to south, showing that such a national-scale product can clearly identify regional variations to help formulate more effective management strategies.

We also presented an innovative method to reconstruct the prehistoric landscape by filling the man-made ditches—essentially virtually restoring them—and recreating the channel network of the prehistoric landscape through modeling. This enabled us to identify the human impacts on the small-scale waterways of the forest landscape. This site-specific, prehistoric change analysis of the channel network could support effective decision-making for the restoration of ditches to either peatland or streams (Hasselquist et al. 2018). For example, if a ditch is located near a prehistoric natural channel, it was likely straightened and should be restored to a stream. If a ditch does not overlap a prehistoric channel, it likely was dug into a peatland, DRIP, or wet mineral soil (Ploum et al. 2018), and could be restored to one of those landforms, thus reducing the impacts of ditch networks on DOC and nutrient export. Therefore, this method could have significant implications for spatial land use planning and decision-making for ecological restoration, in addition to identifying and classifying inland waters under the EU Water Framework Directive (Kallis and Butler 2001).

## Conclusions

The small-scale channel network, either natural or man-made, strongly controls hydrological connectivity; yet small-scale channels are poorly considered in environmental management and ecological restoration activities. This study presented a novel approach for effective detection and mapping of small-scale channel networks at a larger landscape scale by integrating high-resolution LiDAR DEMs, manual digitization, and modeling. Through the modeling and mapping of prehistoric and current channel networks, this research produced a comprehensive understanding of the distribution and location of man-made ditches in Sweden that showed a striking human alteration of the hydrological connectivity in the Swedish landscape. Our analysis included 11 selected study regions spanning a north–south gradient across Sweden and concluded that 58% of the prehistoric natural channel network had been converted to various types of ditches. The current landscape of these 11 regions is overwhelmingly dominated by man-made channels—6% agricultural, 25% road, and 56% forest ditches, while the natural channels currently constitute just 13% of the network. This indicates a striking human alteration of the hydrological connectivity in the Swedish landscape. We found that ditching is the largest man-made alteration of the Swedish natural landscape and likely has significantly impacted the soil, hydrology, and forest ecosystems as well as GHG balances. Our new methods can be used to inform site-specific land management, prioritize ecological restoration of wetlands, and improve hydrological monitoring in Sweden. Additionally, the methodology can be implemented in any northern landscape for understanding the extent of human modification of natural channel networks to guide future environmental management activities and policy formulation. Our study regions were dominated by forests; therefore, future analyses that include agricultural lands and protected wetlands could be more comprehensive in explaining the impacts of ditching on multiple ecosystem processes. Altogether, man-made ditches can have profound impacts on several ecosystem processes, and hence, detection, accurate mapping, and monitoring of the ditch network are critical for effective ecosystem management.
